# Uniformity in Hospital Accounts

**Published:** 1887-02-26

**Authors:** F. Browne

**Affiliations:** Secretary of Isle of Man Hospital


### THE EDITOR'S LETTER-BOX.

[Our Correspondents are reminded that prolixity is a great bar to publication, and that brevity of style and conciseness of statement greatly facilitate early publication.]

### UNIFORMITY IN HOSPITAL ACCOUNTS.

WILL you kindly send me a printed form of return containing all the particulars to be filled up by competitors for your Quilter Prizes? I do not intend to compete; but should like to have the form, in order that, if practicable, the accounts of the Isle of Man Hospital (of which I am Secretary) may in future be kept analysed under the several heads which the form may contain. At present our accounts are to some extent analysed, but not so minutely as they might be. May I suggest that if one of your forms were sent to the secretary or treasurer of each provincial hospital, with a request to keep their accounts separated under the various head given in it, the result would probably be to enable you and others more readily to obtain uniform returns, and consequently information of greater value for purposes of comparison?

F. BROWNE,
Secretary of Isle of Man Hospital.

*** This is an excellent suggestion, which the Hospitals Association have determined to put into practice at once. We hope Mr. Browne will compete, as it would be interesting to note how the Isle of Man Hospital compares with others in London and elsewhere.—ED. T. H.

